# A narrative review of estimands in drug development and regulatory evaluation: old wine in new barrels?

**DOI:** 10.1186/s13063-020-04546-1

**Published:** 2020-07-23

**Authors:** M. Mitroiu, K. Oude Rengerink, S. Teerenstra, F. Pétavy, K. C. B. Roes

**Affiliations:** 1 Methodology Working Group, College ter Beoordeling van Geneesmiddelen, Graadt van Roggenweg 500, 3531 AH Utrecht, The Netherlands; 2 Department of Biostatistics and Research Support, Clinical Trial Methodology, Julius Center for Health Sciences and Primary Care, University Medical Center Utrecht, University of Utrecht, Heidelberglaan 100, 3584 CX Utrecht, The Netherlands; 3 Department for Health Evidence, Section Biostatistics, Radboud Institute for Health Sciences, Radboud University Medical Center, Geert Grooteplein 21, 6525 GA Nijmegen, The Netherlands; 4 Biostatistics and Methodology Support Office, European Medicines Agency, Domenico Scarlattilaan 6, 1083 HS Amsterdam, The Netherlands

**Keywords:** Estimand, Intention-to-treat, Per protocol, Intercurrent event, Post-randomisation event, Study withdrawal, Treatment compliance, Treatment adherence, Treatment discontinuation, Missing data

## Abstract

**Background:**

An estimand defines the target of estimation for a clinical trial through specification of the treatment, target population, variable, population-level summary and of the strategies for intercurrent events. A carefully defined estimand aligns the clinical trial design and analysis with the scientific question of interest and adequately accounts for so-called intercurrent events. The ICH E9(R1) addendum suggests five estimand strategies. We evaluated to what extent current practice in drug development and regulatory assessment fits in the estimand framework.

**Methods:**

We systematically evaluated what estimands, especially what strategies for intercurrent events are advised in European Medicines Agency disease guidelines, used in sponsors’ trials and additionally requested by the European Medicines Agency in assessment dossiers. We selected four therapeutic areas: nervous system, oncology, cardiovascular diseases and respiratory diseases. For each, we evaluated all disease guidelines with approved drugs, the dossiers of the most recently approved drugs matching the guidelines and corresponding regulatory questions.

**Results:**

Strategies for intercurrent events were present in 18 (53%) of 34 guidelines, in all 34 sponsor documentations and in 15 (44%) of 34 sets of regulatory questions. Treatment policy was advised in 13 (38%) guidelines and was applied in 9 corresponding sponsor documentations. Of these 9, it was the sole strategy in 4 cases and accompanied by another strategy in 5 cases. Hypothetical strategy was not advised in guidelines. However, it was the leading strategy applied in 25 (74%) sponsor documentations. Composite strategy was advised in 3 (9%) guidelines and applied accompanied by another strategy in 2 corresponding sponsor documentations. While on treatment strategy was not advised in guidelines, but was applied in 2 sponsor documentations. Principal stratum strategy was advised in 2 guidelines but not applied in corresponding sponsor documentations. Of the regulatory questions, treatment policy was present in 2 cases (6%), hypothetical in 6 cases (18%), composite in 6 cases (18%) and while on treatment in 1 case (3%).

**Conclusions:**

Estimand attributes are present in guidelines, sponsor documentations and regulatory questions, but not described as estimands. Treatment policy was most often advised in guidelines, but hypothetical was the leading strategy applied in sponsor documentations. Thus, results indicate not a full concordance between the regulatory target of estimation and what is actually estimated. The lack of concordance was mostly due to limitations in collection of intercurrent events data to enable a treatment policy strategy. There is, therefore, a need to better define estimands at the design stage and throughout the applications dossiers and assessment reports.

## Background

In randomised controlled clinical trials, the aim is to estimate the effect of an intervention compared to a control treatment, unconfounded by assignment to intervention or control. Through randomisation, it is intended that any difference in clinical outcome can be attributed to the intervention and can be causally interpreted [1]. In practice, post-randomisation events, such as treatment discontinuation, use of concomitant medication or a switch in treatment arm, may be related to the treatment. These post-randomisation events cause missing outcome values or more complex; they introduce bias in outcomes obtained. They do not preserve randomisation and subsequently do not allow the randomisation-based inference, hence impacting the estimation of the treatment effect and/or its interpretation. In this context, many methods were proposed to deal with missing data, such as mixed models or imputation methods, or using a composite endpoint treating missing values as non-responders in order to minimise bias [2–4]. However, little attention was given to what impact these missing data handling methods actually have on the treatment effect to be estimated at target population level in realistic conditions. There was a fundamental lack of common understanding between involved stakeholders of what these methods aim to estimate in relevant target patient population terms, as well as a lack of harmonisation in applying and interpreting these methods [5].

The ICH E9 ([6]) describes the intention-to-treat (ITT) principle and the analysis set (FAS).

“The intention-to-treat principle implies that the primary analysis should include all randomised subjects. Compliance with this principle would necessitate complete follow-up of all randomised subjects for study outcomes. In practice this ideal may be difficult to achieve, for reasons to be described. In this document the term ‘full analysis set’ is used to describe the analysis set which is as complete as possible and as close as possible to the intention-to-treat ideal of including all randomised subjects.”

Hence, it points out to the reader that in practice, it may not be possible to have all outcomes observed for all randomised patients in order to comply with the intention-to-treat principle. Furthermore, the full analysis set is used to describe the population almost the same as all randomised patients and certain criteria are mentioned (with respect to treatment intake and missing data) that could lead to patients being excluded from the FAS, e.g. “the failure to take at least one dose of trial medication and the lack of any data post randomisation”. However, it does not mention the scenario when some of the randomised patients have only partially observed outcome data, such as in a longitudinal trial with repeated measurements at protocolled visits, that have all visits but the last one at end of trial, irrespective of other post-randomisation events that did not lead to missing data. Therefore, the trialists are in a difficult and challenging position where something has to be done for the patients with partially or fully missing outcomes (e.g. after they discontinue study, regardless of their reason) in order to comply with the intention-to-treat principle and to reach a full analysis set. It is yet unclear what was done or what can be done in order to include these patients in the (m)ITT analysis.

The term “estimand” is not new in statistics [7]. More recently, it was used as a solution for the “missing data problem” [8, 9]. In 2017, it was incorporated into ICH E9(R1) draft addendum on estimands and sensitivity analysis in clinical trials ([10, 11]), primarily to precisely define the treatment effect in a randomised trial. This addendum supplements the ICH E9 guideline “Statistical Principles for Clinical Trials” ([6]) from the International Council for Harmonisation of Technical Requirements for Pharmaceuticals for Human Use (ICH) [12]. The addendum recommends that the estimand should be precisely defined upfront, which addresses more than the “missing data problem”.

The draft E9(R1) addendum defines four attributes to describe the estimand: variable (or outcome), population, population-level summary and strategies to account for intercurrent events.

*The variable* (or outcome) to be obtained or measured for each individual patient that is required to address the scientific question. If we use an example from pain medication, the variable could be a visual analogue score (VAS) obtained at pre-specified visit times in a trial for acute pain treatment, e.g. VAS to be measured or obtained at baseline, at week 4, week 8 and at week 12 (end of trial).

*The population*, referring to the patients targeted by the scientific question. In the trial for pain treatment, the population could be “adults suffering from acute pain”.

*The population-level summary* for the variable which provides a basis for a comparison between treatment conditions. For example, it could be the difference in VAS means between the experimental and control arm at a pre-planned timepoint, e.g. at 12 weeks.

*The specification of how to account for intercurrent events* to reflect the scientific question of interest (through strategies for intercurrent events); intercurrent events are defined in E9(R1) addendum as “events occurring after treatment initiation that affect either the interpretation or the existence of the measurements associated with the clinical question of interest.” The E9(R1) addendum suggests five strategies to address intercurrent events: (1) “treatment policy”, (2) “hypothetical”, (3) “composite”, (4) “while on treatment” and (5) “principal stratum”. For instance in the trial for pain treatment, self-administration of additional medication for pain might be prohibited by the protocol, but some patients do take it. With a treatment policy strategy, the intercurrent event “need for additional medication for pain” is actively ignored, and the VAS is used as it is for those patients that take additional medication. The treatment policy strategy would technically correspond to the intention-to-treat principle. With a hypothetical strategy, a scenario is envisaged where the intercurrent event “need for additional medication for pain” would not occur. With this strategy for instance, the VAS values following intercurrent event are set to missing if such is in accordance with the hypothetical scenario considered. With a composite strategy, the intercurrent event is explicitly taken into account and made part of the outcome, for instance, by assigning a worst value of VAS, or by considering the patient a non-responder if a binary outcome is used. With a while on treatment strategy, for this intercurrent event takes the form “while no need for additional medication for pain” and VAS values following intercurrent event are not of interest. With a principal stratum strategy, based on baseline covariates, the stratum of patients that would not experience the intercurrent event is tried to be identified. Analysis is then conducted on this stratum. The addendum informs that principal stratum should be distinguished from any type of analysis in a subgroup of patients, such as per protocol or complete case analysis. The E9(R1) addendum also describes scenarios with two different intercurrent events handled by the same strategy or each of the two intercurrent events handled by a different strategy.

The final version of the ICH E9(R1) addendum was published in December 2019 and uses five attributes [13]. One of the five attributes from the final addendum, the “treatment”, was added compared to the four attributes of the draft addendum. The strategies for intercurrent events and their definitions are not different between the draft and the final versions of the addendum. The other three attributes were slightly restructured. In the remainder of this article, we followed the structure and the four attributes from the draft version of ICH E9(R1) addendum.

It was expected that the estimand was not defined explicitly in the terms of these attributes in protocols and reports before publication of the draft addendum. However, clinical trials still had a primary objective with a primary outcome variable, a target of estimation at population level and a pre-specified statistical analysis. This entails that to some extent and at least implicitly, the key elements of an estimand are expected to be present in clinical trials before the E9(R1) addendum concepts became public.

A survey published in 2017 found that an intention-to-treat estimand was most often aimed at and that the most often used methods for missing data handling were mixed-models repeated measures (MMRM) or last observation carried forward (LOCF) imputation [14]. In the precise language of E9(R1), there is likely a mismatch between the aim of intention-to-treat (“treatment policy”) and these often used methods of dealing with missing data. Hence, impact of implementation on design and analysis of trials can certainly be expected, but it is currently unclear how large the impact of the proposed estimand framework may be. It is important to identify to what extent the framework leads to different effect estimates compared to current practice in drug development and regulatory assessment. We therefore aimed to answer the following research questions:

1. What types of estimands, especially what strategies to account for intercurrent events, are advised in European Medicines Agency (EMA) disease guidelines?
2. What types of estimands, especially what strategies to account for intercurrent events, are used by sponsors in their confirmatory clinical trials supporting the application for marketing authorisation?
3. What additional types of estimands, especially what strategies to account for intercurrent events, are requested by the regulatory agencies in reply to the sponsor documentations in assessment dossiers?

## Methods

We systematically evaluated what estimands were targeted in regulatory disease guidelines, in trials from recently approved applications and in regulatory questions. We scrutinised what strategies to account for intercurrent events were advised, used and further requested in drug development and evaluation. We performed this review on EMA ([15]) disease guidelines ([16]) and on corresponding approved medicinal products dossiers [17].

### Selection of disease guidelines and medicinal products for evaluation

First, we selected all EMA disease guidelines (described hereafter as the “guidelines”) within four therapeutic areas: nervous system, oncology, cardiovascular diseases and respiratory diseases, to identify the diseases for which regulatory guidance is available for clinical efficacy investigation. We considered these four main therapeutic areas to have the broadest coverage of most estimands practices.

**Fig. 1:**
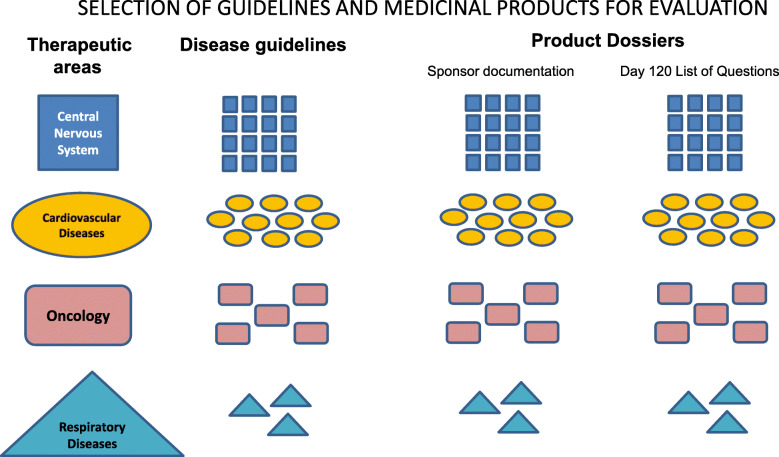
Illustration of the process employed for data extraction and interpretation

**Fig. 2:**
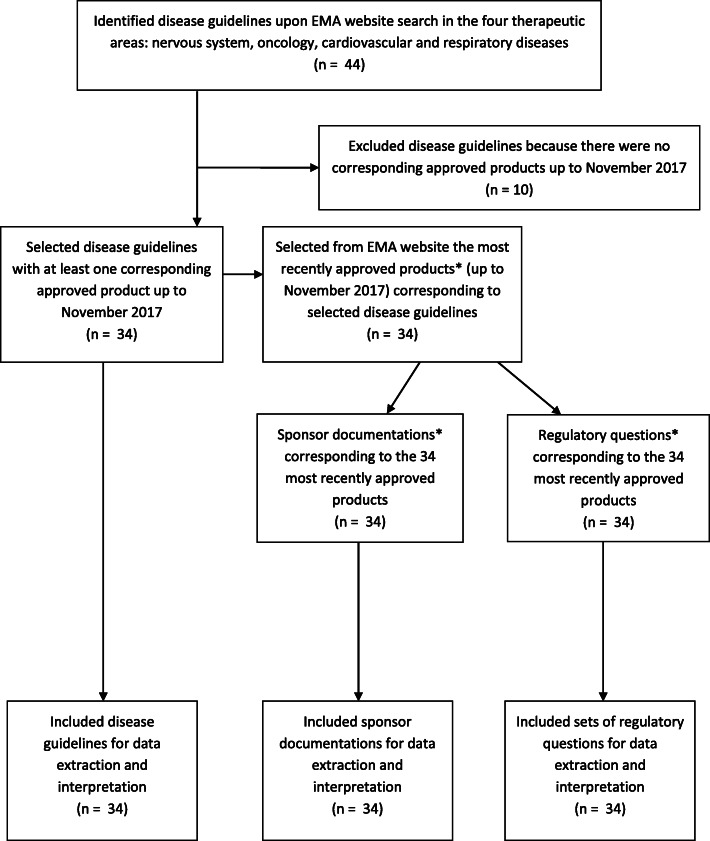
Flow diagram detailing the identification, selection and inclusion of disease guidelines and approved products for data extraction and interpretation. *one approved product= one sponsor documentation and one corresponding set of regulatory questions

In November 2017, for all identified diseases within these four therapeutic areas for which regulatory guidance is available, we selected the most recently approved innovative product in the centralised procedure (Figs. 1 and 2), defined by the date of positive opinion from the Committee for Medicinal Products for Human Use (CHMP) [18]. The most recently approved products were assumed to best reflect current practice. We limited our selection to one product within each disease as a snapshot of how the estimands principles were employed in practice. We excluded guidelines for which there was no approved product available up to November 2017. We used the version of the guideline that was effective at the time of approval for each particular medicinal product, with a few exceptions. In case a new guideline became effective closely after product approval, it was assumed that draft information was available through the public consultation phase, therefore impacting already clinical trial design in practice.

For each selected product, we used the sponsor’s protocols, statistical analysis plans and clinical study reports (altogether described hereafter as the “sponsor documentation”) for the confirmatory clinical trials supporting the application for authorisation. We extracted the questions raised by the EMA during the assessment procedure verbatim from the “day 120 list of questions” of the centralised procedure (described hereafter as the “regulatory questions”). This list of questions is expected to capture the most extensive and least selective list of efficacy-related questions raised by the EMA. Regulatory questions contain the “major objections” and the “other concerns”, which can be supplementary questions addressed by regulators to the sponsor, based on the evidence provided in the application dossier which includes the sponsor’s documentation [19].

### Data extraction

We used three sources of information for data extraction: the guidelines, the sponsor documentation and regulatory questions. We extracted from each data source all relevant phrases and paragraphs pertaining to the target of estimation (estimand and its attributes as per ICH E9(R1) draft addendum) corresponding to the primary analyses and corresponding supplementary/sensitivity analyses:

- Variable/outcome,
- Population,
- Factors that are likely to influence the treatment effect (e.g. rescue medication) (potential intercurrent events) and information regarding the missing data handling,
- Comparison (statistical contrast) upon which treatment effect is interpreted, called population-level summary in the ICH E9(R1) addendum.

For each selected disease guideline, we used the most recent version from the EMA website. For each selected medicinal product, we used the dossiers that we retrieved from the document management system of CBG-MEB and EMA database. We created a data extraction form, collected and stored the information in Microsoft Office Access Database (Appendix 2).

We pilot-tested the first version of the data extraction form on two products for guideline, sponsor documentation and regulatory questions. Following this pilot test, we refined the form.

### Data interpretation and translation from efficacy-related information to estimand attributes

During the research, it became obvious it is necessary to add a data interpretation step in order to translate the raw, unstructured information derived from the documentation into estimand constructs. This was the case especially for the intercurrent events and strategies to account for them. The information pertaining to variable, population-level summary and target population was more straightforward to map from the basic information.

For each guideline, corresponding sponsor documentation and regulatory questions respectively, we interpreted the estimand attributes in order to reconstruct the corresponding implied estimand.

The E9(R1) draft addendum was not yet published when the guidelines were published, when the trials were conducted or when the regulatory questions were raised; therefore, the attributes were not expected to be phrased and framed in a dedicated section and not in the shape specified in E9(R1) draft addendum. The information had to be translated from text referring to the efficacy analysis into an estimand attribute (e.g. “concomitant medication use” as intercurrent event). For population-level summary, we used the comparison (statistical contrast such as difference in means, odds ratio) on which the treatment effect quantification and/or magnitude is assessed and concluded on. This was typically derived from the proposed primary analysis. For population, we used the population description in analyses or in analyses sets. The variable could be extracted as it was described. We categorised the attributes as “present” or “not present”. The attributes are qualified as “present” if they fulfil the above definitions as per draft E9(R1), regardless of where in the documents the information was found. In the example below, the attributes were considered “present”.

**Table:** 

Attribute	Variable	Population	Strategy to account for the intercurrent event	Population-level summary
**Phrase verbatim extracted**	“the primary endpoint for the primary analysis is LDL-c”	Inclusion/exclusion criteria + “The FAS comprised all patients that were randomised and had an evaluable outcome value at 12 months”	“patients with missing values that switch regimens or discontinue assigned treatment are counted as failures”	“Difference in mean change from baseline of LDL-c at 6 weeks”

The above extracts are factual quotes as found in the examples reviewed

Conversely, if only non-specific statements, such as “The effect of missing values will need to be taken into account in the efficacy analysis and the method to address this problem needs to be pre-specified”, without being incorporated in the analysis or without clear and explicit instruction, then the intercurrent event attribute is considered “not present”. All other attributes will be qualified as “not present” if they are not specified and cannot be determined given their definitions in E9(R1) draft addendum.

Within intercurrent events, we created the category of intercurrent events “not accounted for” (NAF). This category represents the intercurrent events that were identified and possibly collected, but not included or referred to in the primary efficacy analyses, for example, “concomitant administration of systemic corticosteroids”, “change in background medication” or “salty food intake”.

We determined whether the estimand could be reconstructed from the information provided. If all attributes are scored “present”, then the estimand is classified as “can be determined”. If any of the attributes are deemed “not present”, then the implied estimand will be classified as “cannot be determined”. We interpreted the strategies using the five types of strategies proposed and defined in E9(R1) addendum: treatment policy, hypothetical, composite, while on treatment and principal stratum. Where the strategies type did not fit in one of the E9(R1) definitions, we described the strategy in detail and classified them as “other”. Per protocol analysis was not defined in the addendum, but depending on how it is defined then it could have been correspondent to a strategy, e.g. while on treatment.

To understand the strategies for intercurrent events and reconstruct the estimand, we also extracted information pertaining to the statistical analysis and imputation methods.

### Quality review

The concept of intercurrent events is the novel aspect introduced with the estimand framework. The quality review therefore focused especially on the strategies to account for the intercurrent events, as these needed most interpretation. The other estimand attributes were deemed unambiguous to determine by the primary data extractor and interpreter (primary reviewer) as well as by the secondary reviewers. As we found that guideline texts often led to difference in opinions regarding presence of intercurrent events and strategies to account for them, we proceeded with full double review of all guidelines.

One person (MM—primary reviewer) extracted the data, translated it to estimand attributes and reconstructed the implied estimand. For quality control of all guidelines estimand constructs, two secondary reviewers (ST and KOR) each read the entire guidelines content and reviewed the estimands constructs next to the primary reviewer. Differences were solved in consensus between the primary and secondary reviewers. If consensus could not be reached between the primary and secondary reviewer, a third reviewer would be consulted (KR/FP) and discussed until consensus was reached. Each secondary reviewer performed the quality review for half of the selected set of guidelines.

The sponsor documentations and regulatory questions were less ambiguous to interpret, and we considered the efficacy analyses in general detailed enough in order to adjust the depth of the quality review. Each secondary reviewer performed the quality review independently for five different products dossiers (sponsor documentations and the corresponding regulatory questions). If > 25% of the intercurrent events and implied strategies would not match, then a full review would be triggered for all sponsor documentations and corresponding regulatory questions. If < 25% discrepancies but with systematic or recurrent errors (e.g. consistent mismatch in a particular strategy or combination of strategies), the primary reviewer would re-review all dossiers for that particular error.

### Analysis and summary of results

We summarised the estimands and attributes overall and per therapeutic area. The experimental unit for analysis was considered a guideline, the set of sponsor documentation pertaining to one product or the set of regulatory questions related to the sponsor documentation corresponding to that product, respectively. We created cross-tabulations for attributes (“present” or “not present”), intercurrent events and type of strategy for intercurrent events according to the types proposed in E9(R1) draft addendum. We summarised what strategies for intercurrent events and in which combination they were used with the other attributes to define the estimands. If no strategy or no estimand was specified or described, these were summarised as “strategy not present” or “no estimand present”. Given the nature of the review and summaries, no statistical testing was performed.

## Results

We included 34 guidelines for which products were approved, 34 sponsor documentations for the approved products corresponding to the guidelines and 34 sets of regulatory questions corresponding to the approved products we had selected (Appendix 3). Those were selected from the therapeutic areas nervous system (*n* = 16), oncology (*n* = 5), cardiovascular diseases (*n* = 10) and respiratory diseases (*n* = 3). Guidance documents effective dates ranged from 1992 to 2017, products approval ranged from 1996 to 2017 and regulatory questions dates ranged from 1995 to 2016. Two secondary reviewers agreed without or with limited changes with the data extracted and their interpretation. For the 10 sponsor documentations and regulatory questions that were reviewed in pairs, reviewers agreed more than 75% of extractions (90% with ST, 80% with KOR); hence, full peer review of all sponsor documentations and regulatory questions was not triggered.

### Description of the four estimand attributes

All four estimand attributes were specified in 12% of the guidelines, in all sponsor documentations and in 3% of the regulatory questions (Table 1). We found the information pertaining to attributes scattered in different sections throughout the statistical analysis plans, protocols and clinical study reports. The information pertaining to attributes was easy to find in guidelines, but more difficult to find in sponsor documentations. The attributes were not described explicitly and often embedded in primary efficacy and statistical methods, missing data handling, data collection or results sections. If described, the attributes were found relatively easy in the regulatory questions in the section for clinical efficacy (Appendix 1). However, not all attributes are described explicitly for all analyses requested in the regulatory questions.

**Table 1:** Frequency of attribute presence/description in guidelines, sponsor documentations and regulatory questions

	Frequency of attribute presence		
Source document	Guidelines (***N*** = 34) (%)	Sponsor documentations (***N*** = 34) (%)	Regulatory questions (***N*** = 34^*^) (%)
Attribute			
Variable	100	100	68
Population	24	100	3
Population-level summary	38	100	3
Intercurrent events	79	100	68
Strategies intercurrent events	53	100	44

^*^23 out of 34 regulatory questions documents had estimand-related questions

#### The variable (or outcome)

The variable was present in all 34 guidelines, in all 34 sponsor documentations and in 23 (68%) of 34 sets of regulatory questions.

#### The population

The population was described in 8 (24%) guidelines, in all 34 sponsor documentations and in 1 (3%) set of regulatory questions. An intention-to-treat analysis (ITT corresponding to a treatment policy strategy) is advised in most guidelines.

We found in sponsor documentations multiple ways in which the analysis population deviated from the definition of the intention-to-treat principle [6]. Even if described using the same term “modified intention-to-treat”, the modifications varied between products and studies (Table 2). And, although the term “Intention-to-treat” was used, not all randomised patients were included in the analysis as the ITT principle dictates.

**Table 2:** Variations of intention-to-treat (ITT)

Population description	Name	Formulation(s)
ITT, mITT, FAS	Intention-to-treat, modified intention-to-treat, full analysis set	1. All randomised patients with at least one follow-up measurement available
		2. All randomised patients that took any/at least one dose of trial medication
		3. All randomised patients with the baseline measurement available and at least one post-baseline measurement available
		4. All randomised patients with baseline measurement available, at least one post-baseline measurement and took any/at least one dose of trial medication
		5. All randomised patients with at least one post-baseline measurement and took any/at least one dose of trial medication

#### The population-level summary for the variable

The population-level summary was present in 13 (38%) guidelines, in all 34 sponsor documentations and in 1 (3%) set of regulatory questions.

#### The strategies to account for intercurrent events

Intercurrent events were described in 27 (79%) guidelines, in all 34 sponsor documentations and in 23 (68%) sets of regulatory questions. Strategies to account for intercurrent events were present in just over half of the guidelines (*n* = 18, 53%), in all sponsor documentations (*n* = 34, 100%) and in almost half of the regulatory questions (*n* = 15, 44%) (Table 1).

Treatment policy strategy was advised in 13 (38%) guidelines. It was applied in 9 corresponding sponsor documentations. Of the 9, it was the only strategy applied in 4, and in 5 it was applied accompanied by another strategy or analysis for different intercurrent events. In the four remaining sponsor documentations, a different strategy (or a mix of other strategies) than treatment policy was applied. Treatment policy strategy was applied in total in 13 sponsor documentations; hence, in 4 of these 13 sponsor documentations, it was applied in the absence of being advised in the guideline.

Hypothetical strategy was not advised in any of the 34 guidelines. However, it was the sponsors’ preferred strategy, applied in 25 sponsor documentations, especially to account for missing outcome values (caused by known or unknown intercurrent events). Hypothetical strategy was generally used in the same estimand simultaneously with another strategy, usually with the treatment policy strategy applied for a different intercurrent event. The typical hypothetical strategies were identified in relation to LOCF, MMRM and censoring in time-to-event analysis. These were related to missing data and were used as a measure to explicitly/implicitly impute or handle missing outcomes that were planned to be collected but were not.

Composite strategy was advised in 3 (9%) guidelines. It was applied in 2 corresponding sponsor documentations. Of the 2, it was not applied as single strategy in any of the corresponding sponsor documentations; it was applied accompanied by another strategy or analysis for different intercurrent events. In the one remaining corresponding sponsor documentation, a different strategy (or a mix of other strategies) than composite was applied. Composite strategy was applied in total in 6 sponsor documentations; hence in 4 of these 6 sponsor documentations, it was applied in the absence of being advised in the guideline.

While on treatment strategy was not advised in any guideline but was applied in 2 (6%) sponsor documentations. Clinical outcome (events) was measured over the non-missing days or number of events were adjusted for the treatment period (a negative binomial model with offset for treatment exposure period), both in CNS therapeutic area. The population-level summary was a contrast for rates of events.

Principal stratum strategy was advised in 2 (6%) guidelines but was not applied in any sponsor documentations.

The estimands advised in guidelines and requested in regulatory questions contained a single strategy intending to cover one or multiple different intercurrent events at the same time, such as a treatment policy strategy applied for all intercurrent events. The estimands in sponsor documentations contained multiple strategies to concomitantly handle multiple different intercurrent events at the same time, such as a treatment policy strategy applied for one intercurrent event and hypothetical strategy for another.

In the 16 sponsor documentations corresponding to guidelines where a strategy was not advised, the hypothetical strategy was most often used for some intercurrent events and in conjunction with another strategy for other intercurrent events.

Of the regulatory questions, treatment policy strategy was present in 2 (6%), hypothetical strategy in 6 (18%), composite strategy in 6 (18%), while on treatment strategy in 1 (3%) and principal stratum strategy in none (0%).

**Fig. 3:**
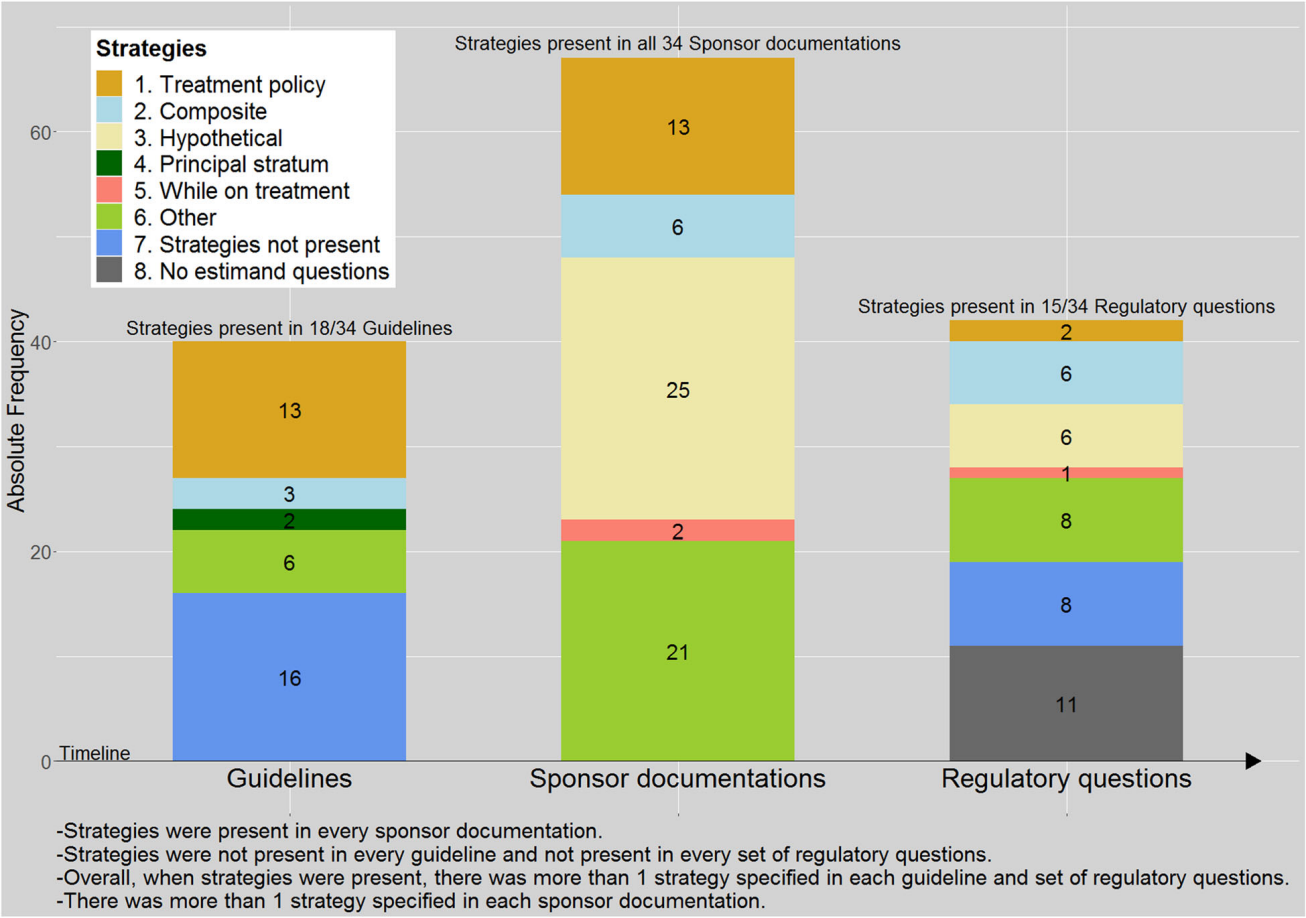
Stacked barplot with strategies in guidelines, sponsor documentations and regulatory questions

Apart from the five strategies suggested and defined in the draft E9(R1), we found other types of analyses that do not fall within any of the five strategies definitions, we summarised these as “other” (Fig. 3). We found them in guidelines, sponsor documentations and regulatory questions. Over half of the “other” category was a per protocol analysis, using various definitions of protocol violations or deviations. Furthermore, we also encountered complete cases or available case analyses. None of these could be usefully categorised as estimand strategy.

#### Strategies by therapeutic area

**Fig. 4:**
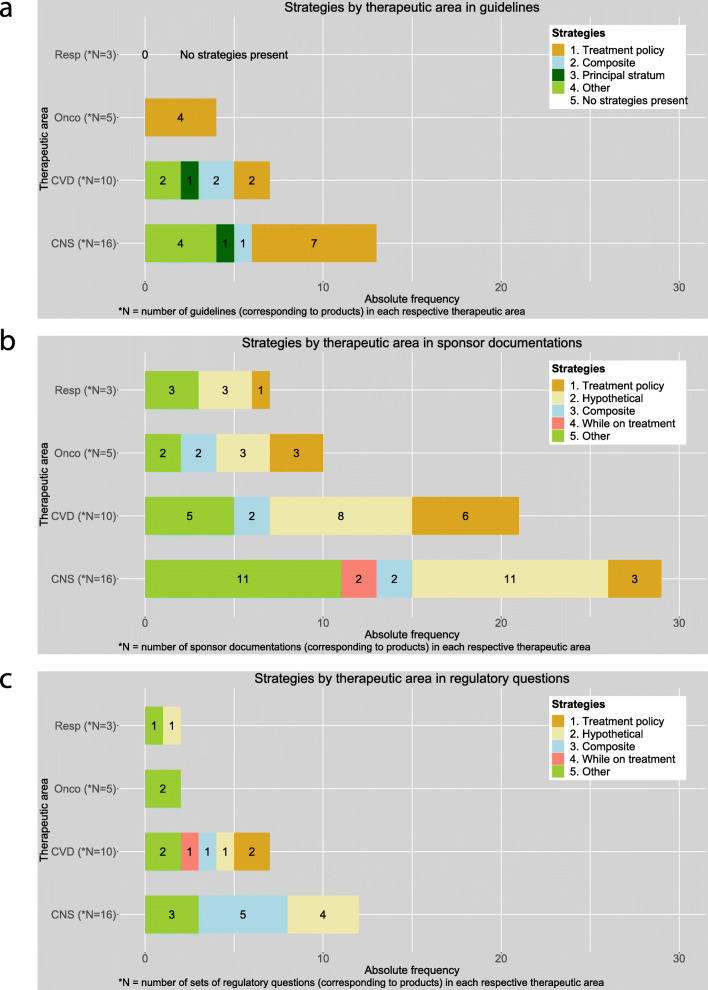
Strategies by therapeutic area in **a** guidelines, **b** sponsor documentations and **c** regulatory questions. CNS, central nervous system; CVD, cardiovascular diseases; Onco, oncology; Resp, respiratory diseases

Treatment policy was the most often advised strategy in guidelines for each therapeutic area. Composite and principal stratum strategies were present in central nervous system and cardiovascular disease guidelines but were not present in guidelines for oncology and respiratory diseases (Fig. 4a).

The estimands suggested in guidelines contained a single strategy covering multiple different intercurrent events at the same time, such as a treatment policy strategy applied for all intercurrent events.

In sponsor documentations, hypothetical strategy was the leading strategy in each therapeutic area, followed by treatment policy strategy. While on treatment strategy was seen only in cardiovascular diseases. All therapeutic areas, except respiratory diseases, used the composite strategy (Fig. 4b).

In sponsor documentations, they usually aimed at a treatment policy estimand. This, however, is often not strictly achievable as per ICH E9 mainly due to limitations in the data. Reported estimands contained multiple different strategies for different intercurrent events or other analyses. For example, they applied within the same analysis treatment policy for some intercurrent event(s) and a hypothetical strategy for other intercurrent events that led to missing data.

Regulatory questions typically contained one estimand (strategy) or analysis per question. The strategies or analyses requested were usually different from a treatment policy strategy, with no clear different pattern between therapeutic areas (Fig. 4c).

## Discussion

With this review, we provide an overview of what the implied estimand practices were in drug development and regulatory evaluation before the publication of the draft version of ICH E9(R1) estimand framework (Fig. 3). Sponsor documentations contained more detailed descriptions of the estimand attributes than guidelines and regulatory questions. A treatment policy strategy was most often advised in guidelines and targeted in sponsor documentations. However, a treatment policy strategy could often not be fully achieved due to incomplete follow-up, resulting in a hypothetical estimand being the most frequent approach by sponsors. Apart from the five strategies defined in the addendum, we also identified other analyses types.

The variable was the estimand attribute most present and clearly defined in guidelines, sponsor documentations and regulatory questions. This was not surprising, as the clinical outcome to be obtained or measured in patients is a pivotal item to decide on, when designing a trial. It already is thoroughly discussed between involved parties. Hence, it is usually described in detail and concordant in all types of documents.

In all sponsor documentations, we found data collected and reported, for example, for drop-out due to adverse events or concomitant medication and these data are used for instance, in safety analyses. However, the included information on intercurrent events (such as an adverse event leading to study withdrawal) was not used nor referred to in the primary efficacy analysis.

We found that strategies advised in guidelines, applied in sponsor documentations and asked for in regulatory questions, were different. There could be several reasons behind this finding; it could be due to the fact that sponsors may have followed advice from disease guidelines under the remit of other regulatory authorities than EMA, such as the FDA guidelines.

Furthermore, guidelines may have advised a single general strategy that cannot be applied for all intercurrent events, such as treatment policy. Sponsors applied the advised strategy for part of the intercurrent events where the advised strategy could be applied, but also had to apply another strategy or analysis for other intercurrent events where the advised strategy could not be applied.

This could be due to lack of precision in articulating the targeted treatment effect in guidelines and also due to feasibility of applying only one strategy given the data and not because of sponsors’ lack of intent to apply advised strategies. It may also be the case that sponsors identified different or more intercurrent events than the ones suggested by guidelines and, hence, based on their treatment effect of interest, decided to apply other strategies than the strategies advised in guidelines. EMA can provide sponsors with scientific advice regarding their trials; however, not all sponsors apply for scientific advice. Moreover, having been provided with scientific advice, as it is not legally binding, it does not make it mandatory for the sponsors to follow it [20].

In spite of sponsor documentations providing more detailed specifications of analyses and estimand-related information, it could often not be inferred from the documentation why certain choices were made for specific intercurrent events. Similarly, the clinical questions raised during the assessment were not phrased precise enough to translate into the intended estimand. Therefore, it was not possible in general to determine how well aligned the estimand was with the clinical question of interest.

Although the strategies in guidelines seem to differ between therapeutic areas, the types of strategies found in sponsor documentations seemed to be similar between therapeutic areas.

The difference between strategies advised in guidelines and implemented by the sponsors could be attributed to several reasons. First, strategies advised in guidelines may not be fully achievable in practice. Secondly, sponsors may have followed disease guidelines and feedback from other regulatory regions, such as from FDA, which might have advised different strategies.

Over half of the “other” category was a per protocol analysis, using various definitions of protocol violations or deviations. We also encountered complete cases or available case analyses. However, none of these yields a proper estimate for a meaningful estimand as the results cannot be generalisable to a broader target population. It is debatable if such an analysis is actually targeting estimates in a principal stratum. It would be at very best an improper analysis for it. In this respect, the draft addendum informs that “treatment effects in principal strata should be clearly distinguished from any type of subgroup or per-protocol analyses where membership is based on the trial data”. To enable analyses in strata, causal inference approaches are likely necessary.

A strength of this research is that this review is the first of this nature. It acts as a snapshot of actual practices with estimands and strategies present in documents pertaining to medicines development and regulatory evaluation. It was done with access to extensive and full documentation of the actual protocols, results and regulatory interaction. It could be used as baseline in future publications following implementation of ICH E9(R1) addendum. We used publicly available guidelines and had access to regulatory agencies databases. Concomitantly with the publication of the E9(R1) addendum and from its perspective, this review provides valuable insights into current estimand practices. Thus, it can facilitate the implementation of the estimand framework in drug development and regulatory evaluation of medicinal products.

Our review also has some limitations. First, there could be other guidelines in other therapeutic areas or within those therapeutic areas for which products were not approved that contain other strategies for intercurrent events. The results observed might not be fully generalisable to those areas. However, this review represents an overview of the estimand practices in the four biggest therapeutic areas. Second, we had to interpret the intercurrent events and strategies to reverse-engineer the estimand. This process was difficult mainly because the clinical question was not detailed and clear enough to understand the targeted treatment effect and the attributes’ information was not structured consistently throughout all documentation. The possible bias due to the interpretation was partly addressed with an independent quality review from two different secondary reviewers which resulted in high percentages of concordance. It is therefore likely that it did not have much impact on the results. Furthermore, we extracted the estimand attributes as defined in the draft addendum and not as in the final addendum. Therefore, we did not collect data for “treatment” attribute. As our attention was focused mainly on the strategies used for intercurrent events, it did not impact our results and conclusions. We were not able to identify clear and unambiguous clinical questions that are addressed in the trials. Most of the times, the clinical trial objective is phrased as “to study the effect of experimental treatment X over control in patients suffering from Y”. We consider this to be insufficiently described, and according to the addendum, this is key to enunciate in detail and adequately. This impacts what treatment or treatment strategy is investigated and directly affects the estimand, regulatory evaluation and approval, and ultimately the label of the medicinal product.

The estimand framework is expected to impact all phases of drug development and regulatory evaluation. Defining the estimand aims to provide clarity and better define the treatment effect in perspective to the question of interest. It will consequently facilitate interaction between regulators, patients, clinicians, investigators, HTA bodies, statisticians and other trialists. Therefore, changes are needed for successful roll-out and alignment with the estimand principles. The estimand framework may not solve the causes of trials issues, such as incomplete patient retention or poor treatment compliance, but it would add clarity on how these can be handled in a transparent and principled manner. Apart from dissemination of the estimand framework in all branches of the medical community by means of training materials, workshops or research articles, another important step might be to update templates of trial protocols. These templates are used at initial stages of development by every involved stakeholder, irrespective of trial type or phase. One such commonly used document where the estimand framework can be introduced is the ICH M11 guideline [21]. This would formalise the need for estimand discussions early in a trial and by all stakeholders. Starting drug development using the estimand framework would ensure that subsequent stages (e.g. study or assessment reports, prescription information for patients etc.) follow the same structure and principles.

In practice, it would be very unlikely that one type of estimand or one type of strategy for intercurrent events would be satisfactory for all stakeholders [22]. For example, a regulatory body might be interested in a treatment policy strategy for an intercurrent event, while a patient would be interested in a principal stratum strategy for the same intercurrent event. It is still to be revealed by further research, how and under which conditions a clinical trial can answer different clinical questions of interest for different stakeholders, with different estimands and/or different strategies for intercurrent events. We suggest to be descriptive and explicit regarding what strategy or strategies are advised, applied or requested, for what intercurrent events. Additional to detailed descriptions, we could use for instance “single-strategy estimand” to define an estimand with one strategy handling one or more intercurrent events at the same time and “multiple-strategies estimand” to define an estimand with two/more different strategies handling two/more different intercurrent events.

Furthermore, we hope the estimand framework is implemented as envisaged in the addendum, to improve the quality with which clinical research questions are addressed by clinical trials. This includes reaching agreement between stakeholders on the estimand(s) of interest, in a transparent, principled and efficient manner.

In ICH E9, the ITT principle is defined [6]. In actual practice, many different deviations from the principle were encountered under the term “modified ITT”. However, any modification to ITT definition based on observed trial data (e.g. patients having to take at least one dose of assigned treatment) may not clearly define an actual targeted population anymore and make results difficult to interpret.

Aiming for an estimand does not guarantee estimability of that estimand from the trial data, and we think this is a common pitfall for interpretation of trials. For instance, treatment policy strategy can be pre-planned to be applied for all intercurrent events but cannot be achieved in full because of missing outcome values for other reasons. ICH E9 informed the reader that the ITT principle may be difficult to achieve as it needs complete follow-up of all randomised subjects for study outcomes [6]. For intercurrent events causing missing data or for missing outcome values, another strategy (e.g. hypothetical or composite) might be applied. Thus, this may lead to an actual estimand that is different from the one aimed at and to answering a question that deviates from the intended clinical question of interest. This situation is encountered in trials and should be acknowledged.

The estimand framework can help in the design of a trial, to pro-actively strike a balance between the estimand aimed for in principle and an estimand that is actually possible to estimate. It will also help revealing the gap between targeted and realised estimands and facilitate discussion among all stakeholders resulting also in a better understanding of drug effect and better comparison across trials or in meta-analysis of clinical trials.

So, are estimands old wine in new barrels? Estimands are both new and old, and missing data as well as intercurrent events in clinical trials are a long existing issue in medical research. Conceptually, it appears the estimands are as “old” as medical research and clinical trials, because it always had estimand elements (e.g. outcome measured) and even if empirically estimated, there was a target of estimation. Estimands in the shape principled by ICH E9(R1) are an innovative solution to deal with fundamental elements of clinical trials, starting from the research question and dealing with intercurrent events, missing data and treatment effect definitions. The estimand framework provides a new framework to align key elements of design, conduct and analysis of clinical trials to adequately answer the clinical question at hand [23].

## Conclusions

Estimand attributes are present in guidelines, sponsor documentations and regulatory questions, but not described as estimands. Treatment policy was most often advised in guidelines, but hypothetical was the leading strategy applied in sponsor documentations. Thus, results indicate not a full concordance between the regulatory target of estimation and what is actually estimated. The lack of concordance was mostly due to limitations in collection of intercurrent events data to enable a treatment policy strategy. There is, therefore, a need to better define estimands at the design stage and throughout the applications dossiers and assessment reports.

## Supplementary information

**Additional file 1.**

**Additional file 2.**

**Additional file 3.**

## Data Availability

The guidelines used in this research are available from European Medicines Agency website: www.ema.europa.eu The dossiers data are not publicly available.
