# Supplementary material for: A narrative review of estimands in drug development and regulatory evaluation: old wine in new barrels?
Source: Trials. 2020 Jul 23;21:671. doi: 10.1186/s13063-020-04546-1 (PMC7376663; doi:10.1186/s13063-020-04546-1)
Supplement: Supplementary file 1 — Additional file 1. [file 13063_2020_4546_MOESM1_ESM.docx]

**Appendix 1**. Structure of information pertaining to estimand attributes and their description

| **Attribute** | **Type of document (Source)** | **Variable/**  **Endpoint** | **Population** | **Population-level summary** | **Analysis/ imputation method** | **Intercurrent events and strategies to account for them** |
| --- | --- | --- | --- | --- | --- | --- |
| **Relevant section from corresponding source used for data extraction** | **Guideline** | - Entire guideline content | - Entire guideline content | - Entire guideline content | - Entire guideline content | - Entire guideline content |
|  | **Sponsor documentation** | - Study objectives - Primary objective - Study design - Primary efficacy - Statistical Methods - Supportive Analyses on the Primary Efficacy Endpoint | - Study objectives - Primary objective - Study design - Inclusion/exclusion criteria - Primary efficacy - Statistical Methods - Supportive Analyses on the Primary Efficacy Endpoint - Study subjects | - Study objectives - Primary objective - Study design - Primary efficacy - Statistical Methods - Supportive Analyses on the Primary Efficacy Endpoint | - Study design - Primary Efficacy - Statistical Methods - Supportive Analyses on the Primary Efficacy Endpoint | - Prior and Concomitant Therapy and Diseases - Prohibited Therapies - Rescue Medication - Prior and Concomitant Diseases - Contraindications - Precautions - Treatment Compliance - Termination of Study - Primary Efficacy - Statistical Methods - Study subjects - Supportive Analyses on the Primary Efficacy Endpoint - Protocol Violations and Deviations - Discontinuations (tables) - Results (tables) |
|  | **Regulatory questions** | - All clinical efficacy MOs and OCs* | - All clinical efficacy MOs and OCs | - All clinical efficacy MOs and OCs | - All clinical efficacy MOs and OCs | - All clinical efficacy MOs and OCs |

^*^Regulatory questions are major objections (MO) and other concerns (OC).
