# Supplementary material for: A narrative review of estimands in drug development and regulatory evaluation: old wine in new barrels?
Source: Trials. 2020 Jul 23;21:671. doi: 10.1186/s13063-020-04546-1 (PMC7376663; doi:10.1186/s13063-020-04546-1)
Supplement: Supplementary file 2 — Additional file 2. [file 13063_2020_4546_MOESM2_ESM.docx]

**Appendix 2.** Data extraction and interpretation form

**Summary conclusion**: [i.e. Attributes not specified apart from the endpoints (many), of interest are IEs concomitant medication and change in background medication, not specified how to be handled, only suggested that these should be clearly thought of in advance and pre-specified in the analysis.]

[Relevant verbatim extracts from each corresponding type of document, corresponding to estimand attributes, statistical analysis and imputation methods.]

**Data collection and handling: []**

**MOV: []**

**NAF**: [i.e. adverse events, used only in safety analyses.]

**PVs: []**

**Other IEs of interest: []**

MOV=missing outcome values, NAF=not accounted for, PV=protocol violation

| **Data extraction** | | | | **Data interpretation** | |
| --- | --- | --- | --- | --- | --- |
| **Variable/**  **endpoint** | **Population** | **Population-level summary** | **Analysis/imputation method** | **Implied intercurrent events** | **Implied strategy/ies**  **to account for intercurrent events** |
| **[]** | **[]** | **[]** | **[]** | **[]** | **[]** |
