# Supplementary material for: A narrative review of estimands in drug development and regulatory evaluation: old wine in new barrels?
Source: Trials. 2020 Jul 23;21:671. doi: 10.1186/s13063-020-04546-1 (PMC7376663; doi:10.1186/s13063-020-04546-1)
Supplement: Supplementary file 3 — Additional file 3. [file 13063_2020_4546_MOESM3_ESM.docx]

**Appendix 3.** Disease guidelines and corresponding products

| **No.** | **Disease guideline** | **EMA Document number** | **Product name** |
| --- | --- | --- | --- |
| 1 | Pain | CPMP/EWP/252/03 Rev. 1 | Prialt |
| 2 | Bipolar disorder | CPMP/EWP/567/98 | Adasuve |
| 3 | Amyotrophic lateral sclerosis | CPMP/EWP/565/98 | Rilutek |
| 4 | Attention deficit hyperactivity disorder (ADHD) | EMEA/CHMP/EWP/431734/2008 | Intuniv |
| 5 | Duchenne and Becker muscular dystrophy | EMA/CHMP/236981/2011, Corr. 11 | Translarna |
| 6 | Multiple sclerosis | CHMP/771815/2011 Rev. 2 | Ocrevus |
| 7 | Depression | CPMP/EWP/518/97, Rev. 1 | Xeristar |
| 8 | Epileptic disorders | CHMP/EWP/566/98 Rev.2/Corr | Briviact |
| 9 | Parkinson’s disease | CHMP/330418/2012 Rev. 2 | Ongentys |
| 10 | Generalised anxiety disorder | CPMP/EWP/4284/2002 | Xeristar |
| 11 | Schizophrenia | EMA/CHMP/40072/2010 Rev. 1 | Reagila |
| 12 | Alcohol dependence | EMA/CHMP/EWP/20097/2008 | Selincro |
| 13 | Smoking cessation | Doc. Ref. CHMP/EWP/369963/05 | Champix |
| 14 | Alzheimer’s disease and other dementias | Doc. Ref. CPMP/EWP/553/95 Rev. 1 | Ebixa |
| 15 | Insomnia | Publication available at <https://onlinelibrary.wiley.com/doi/pdf/10.1111/j.1600-0773.1992.tb00464.x> | Circadin |
| 16 | Acute stroke | CPMP/EWP/560/1998 | Lixiana |
| 17 | Chronic heart failure | CPMP/EWP/235/95 Rev. 1 | Entresto |
| 18 | Hypertension | CPMP/EWP/238/95 Rev. 2 | Edarbi |
| 19 | Lipid disorders | EMA/CHMP/748108/2013 | Praluent |
| 20 | Pulmonary arterial hypertension | CHMP/EWP/356954/08 | Uptravi |
| 21 | Atrial fibrilation | EMA/CHMP/EWP/213056/2010 | Brinavess |
| 22 | Treatment of VTE | CPMP/EWP/563/98 | Lixiana |
| 23 | Acute coronary syndrome | CPMP/EWP/570/98 | Kengrexal |
| 24 | Myocardial infarction | CPMP/EWP/967/01 | Efient |
| 25 | Coronary Artery Disease - angina pectoris (the stable angina) | CPMP/EWP/234/95 Rev.1 | Ranexa |
| 26 | Peripheral-arterial occlusive disease | CPMP/EWP/714/98 rev 1 | Brilique |
| 27 | Asthma | CHMP/EWP/2922/01 Rev.1 | Cinqaero |
| 28 | COPD | EMA/CHMP/483572/2012 | Incruse |
| 29 | Cystic fibrosis | Doc. Ref. EMEA/CHMP/EWP/9147/2008-corr* | Orkambi |
| 30 | Anticancer appendix 4 - NSCLC | EMA/CHMP/703715/2012 Rev. 2 | Tecentriq |
| 31 | Anticancer appendix 4 - prostate cancer | EMA/CHMP/703715/2012 Rev. 2 | Tookad |
| 32 | Anticancer appendix 4- chronic myeloid leukaemia | EMA/CHMP/703715/2012 Rev. 1 | Tasigna |
| 33 | Anticancer appendix 4- myelodysplastic syndrome | EMA/CHMP/703715/2012 Rev. 1 | Vidaza |
| 34 | Anticancer appendix 4- hematopoietic stem cell transplantation | EMA/CHMP/703715/2012 Rev. 1 | Zalmoxis |
